# Basal cell adenocarcinoma of the nasopharyngeal minor salivary glands: a case report and review of the literature

**DOI:** 10.1186/s12885-018-4803-x

**Published:** 2018-09-10

**Authors:** Jia Jin, Xia-Yun He

**Affiliations:** 1 Department of Medical Oncology, Fudan University Shanghai Cancer Center, Shanghai, 200032 People’s Republic of China; 2 Department of Radiation Oncology, Fudan University Shanghai Cancer Center, 270 Dong An Road, Shanghai, 200032 People’s Republic of China

**Keywords:** Basal cell adenocarcinoma, Nasopharynx, Radiotherapy, Minor salivary glands

## Abstract

**Background:**

Basal cell adenocarcinomas (BCACs) arise from the minor salivary glands in the upper respiratory tract and are extremely rare. In this report, we present an unusual case of a 57-year-old male with BCAC that arose from the nasopharynx. To our knowledge, this is the first case report of nasopharyngeal BCAC.

**Case presentation:**

In August 2010, a 57-year-old Chinese male presented with epistaxis and decreased hearing for 1 month. He was diagnosed with BCAC of the solid type that arose from the nasopharynx. The patient received radiotherapy alone and exhibited a complete response. A follow-up at 72 months did not detect any evidence of disease recurrence or metastasis. A comprehensive literature review revealed only 7 previously reported cases of BCAC in the upper respiratory tract. Surgery is the first choice to treat BCAC but may impair maxillofacial function. Radiotherapy is reserved for inoperable cases.

**Conclusions:**

Radiotherapy can achieve good local control and preserve maxillofacial function; therefore, this treatment may be a suitable option for patients who are not good candidates for surgery.

## Background

Basal cell adenocarcinoma (BCAC) of the salivary gland is a rarely diagnosed subtype of all salivary gland neoplasms (1.6%), representing 2.9% of all malignant neoplasms [1]. In the latest World Health Organization (WHO) classification, BCAC was defined as a low-grade salivary malignancy with favourable prognosis [2, 3]. Most BCACs arise from the parotid gland (> 80%), whereas others originate from the sublingual and submandibular glands. Minor salivary glands are most frequently found in the buccal mucosa and hard palate and are less commonly found in BCAC. The involvement of the upper respiratory tract is rare [4–6]. In this report, we present an unusual case of a 57-year-old male with BCAC that arose from the nasopharynx. We describe the clinicopathological features, the immunophenotype and the treatment choices with a review of several case reports in the literature. To our knowledge, this was the first case of BCAC that arose from the minor salivary glands in the nasopharynx.

## Case presentation

**Fig. 1:**
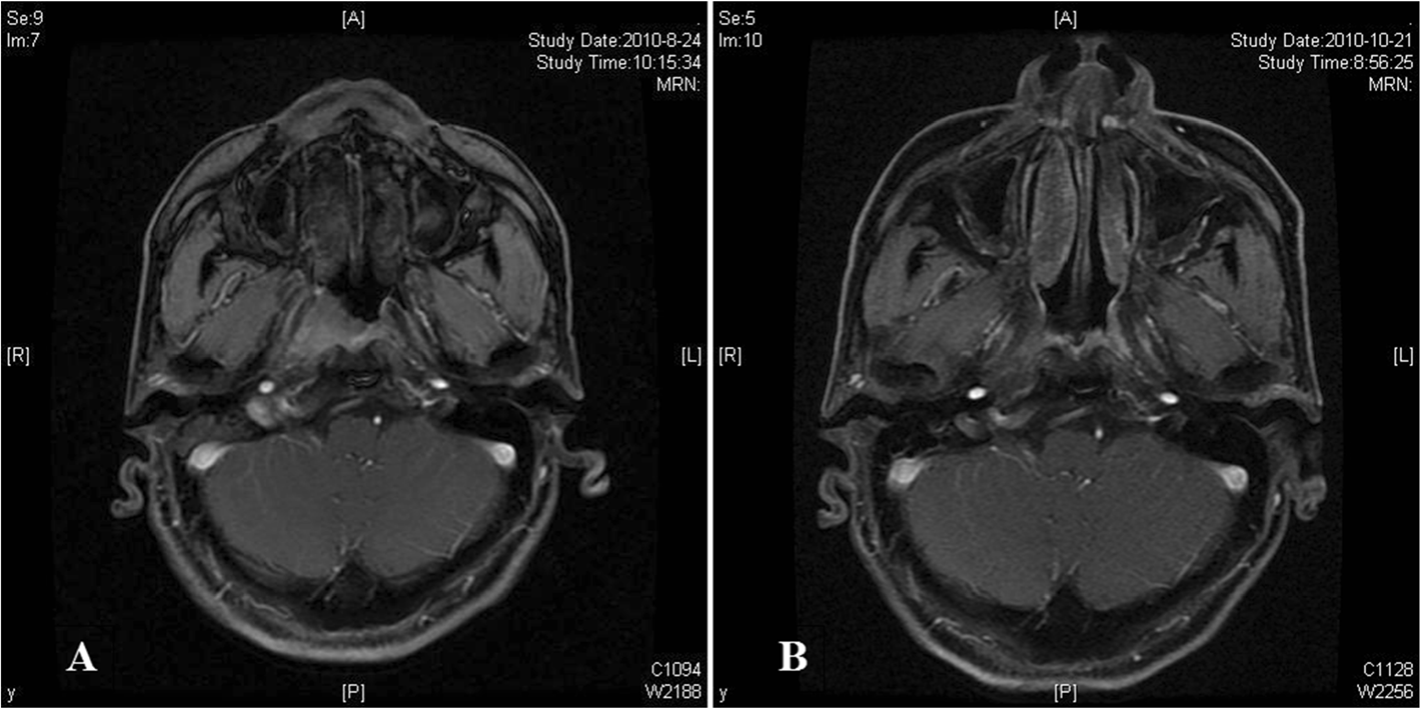
Axial contrast-enhanced MRI scan. **a**, MRI scan (2010.8.14) reveals a tumour in the right posterior nasopharynx that extends into the right parapharyngeal space and into the medial pterygoid muscle. **b**, MRI scan after radiotherapy (2010.10.21) reveals no residual tumour at the primary site

In August 2010, a 57-year-old Chinese male presented with epistaxis and decreased hearing for 1 month. No additional symptoms, such as a neck mass, nasal obstruction, headache, diplopia or other cranial nerve palsies, were noted. The patient had no history of previous or synchronous tumours or any family history of cancer. Nasopharyngoscopy revealed a large exophytic tumour that was covered by smooth mucosa, which grew from the right posterolateral nasopharyngeal wall in the right posterior naris. Magnetic resonance imaging (MRI) scans of the nasopharynx and neck using gadolinium enhancement demonstrated a 2.0 × 1.5 × 2.0 cm well enhanced mass over the right posterior nasopharynx with right retropharyngeal node enlargement. The tumour extended across the right parapharyngeal space and infiltrated into the medial pterygoid muscle. In addition, skull base erosion was detected with right alar lamina involvement (Fig. 1a). Cervical lymph node metastasis was not observed. Hematologic, hepatic and renal function tests revealed no abnormalities. The patients underwent chest and abdomen computed tomography (CT) as well as a bone scintigram, and no distant metastasis was found. A biopsy of the nasopharynx was performed.

In the biopsy specimen, normal salivary tissue was not present. The tumours were ill demarcated without encapsulation. Tumour cells were arranged in nests and nodules. Two morphologic patterns of the tumour cells were observed. Some small round cells exhibited dark nuclei and scant cytoplasm. Other large cells contained round to oval pale nuclei and eosinophilic to amphophilic cytoplasm. In the central region of the tumour cell nests, large cells displayed a solid growth pattern. Small dark cells were clustered at the periphery of the tumour cell nests and appeared palisaded. Prominent nucleoli and mitosis can be observed, and an average of three mitotic figures were observed per 10 high-power fields (original magnification × 400).

**Fig. 2:**
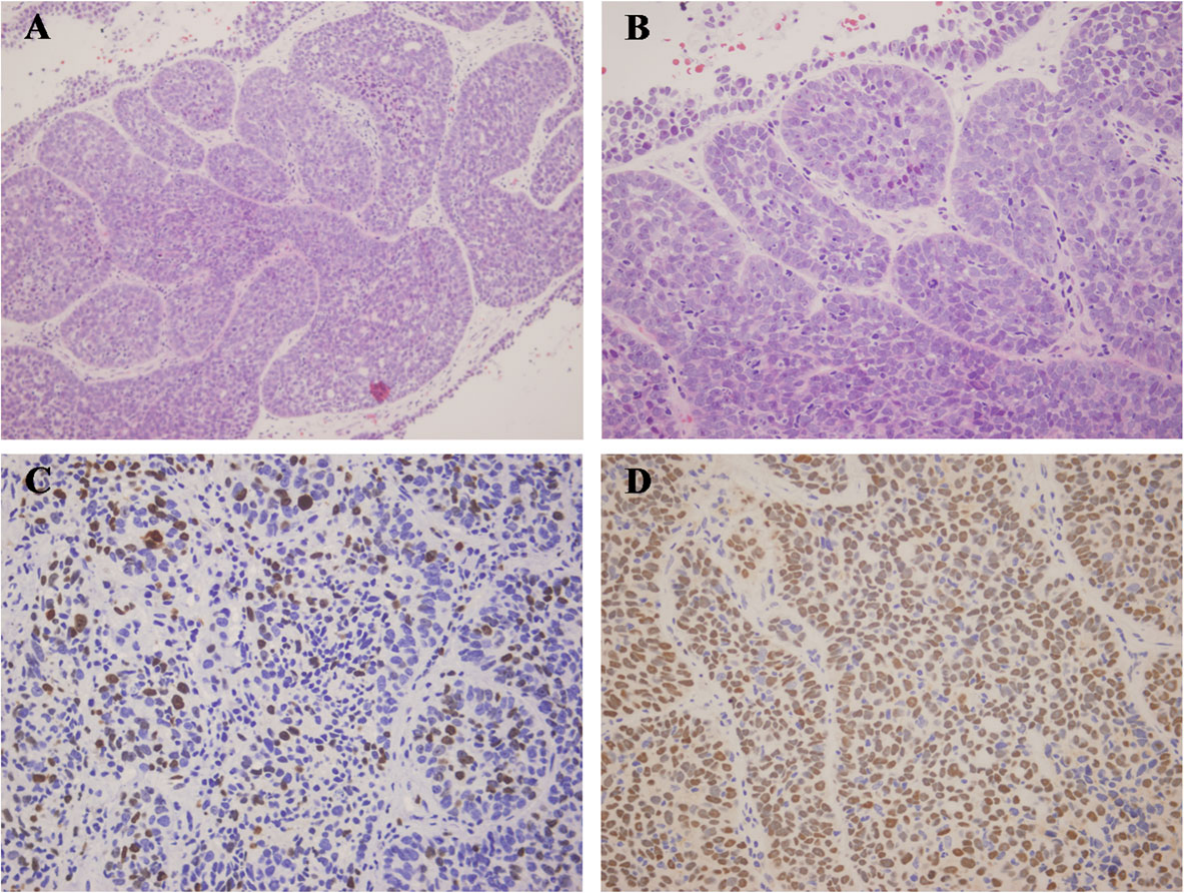
Haematoxylin-eosin stain and immunohistochemical studies. **a**, BCAC revealed solid tumour nests with peripheral palisading (haematoxylin and eosin, original magnification × 200). **b**, The tumour cells exhibited apparent cellular pleomorphism and mitotic figures (haematoxylin and eosin, original magnification × 400). **c**, Immunohistochemical staining for Ki-67 demonstrated a proliferative index of approximately 10% (original magnification × 400). **d**, P63 was strongly expressed by most tumour cells (original magnification × 400)

In the immunohistochemical analysis, the tumour cells were immunoreactive with P63, vimentin, and cytokeratin (CK7 and CK14) antibodies and focally immunoreactive with a calponin antibody. This case of BCAC was not positive for smooth muscle actin or CD117. The proliferative index as demonstrated by Ki-67 was approximately 10%. Based on the immunohistochemistry results and the pathological findings, which included tumour islands with solid proliferation, basaloid-like cells containing large pale and small dark cells, an infiltrative margin, cellular and nuclear pleomorphism, and prominent mitosis, the patient was diagnosed with a solid-type minor salivary gland BCAC (Fig. 2).

Based on the 2002 American Joint Committee on Cancer (AJCC) Tumor, Node, Metastasis (TNM) staging system [7], the tumour was classified as stage III (T3N0M0).

**Fig. 3:**
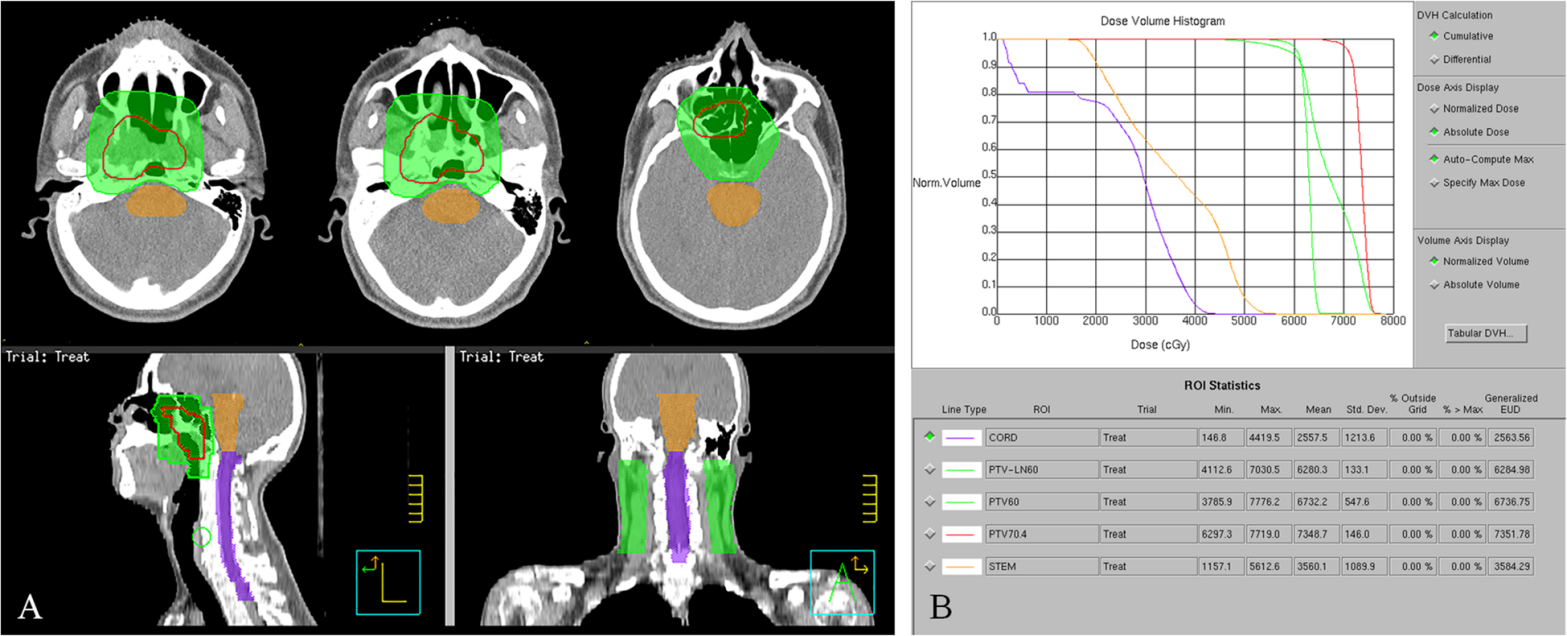
**a**, Planning target volume of the gross tumour volume and the high-risk clinical tumour volume. Axial, sagittal and coronal slices representative of this patient’s planning target volume (PTV) are presented. Red contour represents PTV of the gross tumour volume to 70.4 Gy, green contour represents PTV of the high-risk clinical tumour volume to 60 Gy, orange contour represents brain stem and purple contour represents spinal cord. **b**, Dose volume histogram. The dose volume histogram of this patient is presented. The maximum point dose of spinal cord and brain stem are well within the dose constraint. The prescribed dose encompasses at least 95% of the planning target volume (PTV). None of the PTV receives ≥110% of the prescribed dose

In our case, the patient received intensity-modulated radiation therapy (IMRT) with 6 MV X-rays. The delineation of the gross tumour volume (GTV) was based on the primary tumour volume determined from the physical and imaging examinations. The clinical target volume (CTV) was defined as the whole nasopharyngeal cavity, the clivus, the skull base, the pterygoid plates, the parapharyngeal space, the sphenoid sinus, the posterior one-third of the nasal cavity, the maxillary sinus, and the drainage of the upper neck (levels II, III, and Va. A total dose of 70.4 Gy/32 F/6.2 W was administered based on the planning target volume (PTVg) (GTV with 0.5 cm margin). The PTV60 was defined as 60 Gy/30 F (CTV with 0.5 cm margin) (Fig. 3). After radiotherapy, MRI and nasopharyngoscopy revealed complete disappearance of the tumour (Fig. 1b). The patient was followed up every 3 months for the first 2 years, every 6 months for another 3 years, and then every 12 months. A follow-up at 72 months did not detect any evidence of disease recurrence. The patient developed moderate mucositis as an acute adverse event. However, he did not exhibit any grade 3/4 late adverse events, such as xerostomia, dysgeusia, or hearing impairment.

## Discussion

BCAC that arises from the minor salivary glands in the upper respiratory tract is recognized as an uncommon to rare neoplasm. To our knowledge, only 7 cases have been described in the literature to date [8–10]. Our case is the first case of nasopharyngeal BCAC (Table 1). According to the data in the literature and in this case, this type of tumour predominantly occurs in patients 43–70 years of age with a mean age of 59 years, and no gender differences (a male-to-female ratio of 1.7:1) were observed.

**Table 1:** Studies of BCAC of the minor salivary glands in the upper respiratory tract

Source	Age	Gender	Tumor location	Tumor Size (cm)	Histopathological subtype	Initial treatment	Follow-up	Recurrence	Metastasis	Outcome
Fonseca and Soares, 1996 [8]	62	F	Nasal cavity	NA	Solid	S	4 yrs	Yes	Yes (lymph node)	DOD
	43	F	Maxillary sinus	NA	Solid	S	3 yrs., 6 mos	Yes	No	DOD
	66	M	Maxillary sinus	NA	Solid	S	3 yrs	No	No	Alive
	56	F	Nasal cavity	NA	Solid	S	12 yrs	Yes	No	Alive
	52	M	Ethmoid sinus	NA	Solid	S	12 mos	Yes	No	Alive
Warrick et al., 2000 [9]	66	M	Nasal septum	NA	Tubular	S + RT	1 yrs., 6mos	No	No	Alive (NED)
Jingu et al., 2010 [10]	70	M	Ethmoid sinus	5.0	NA	RT	1 yrs., 9 mos	No	No	Alive (NED)
Present case	57	M	Nasopharynx	2.0	Solid	RT	6 yrs	No	No	Alive (NED)

*F* female, *M* male, *NA* not available, *S* surgery, *RT* radiation therapy, *NED* no evidence of disease, *DOD* die of disease

Neoplasms with a minor salivary gland origin that arise within the nasopharynx include nasopharyngeal carcinoma (NPC), adenoid hypertrophy, lymphoma, fibroangioma, chordoma, and metastatic tumours. NPC is rarely diagnosed in Western Europe and the United States; however, it is more popular in southern China, southeast Asia, northern Africa, and Eskimo areas. In the southern regions of the Chinese mainland, the incidence has been estimated as 30 cases/100,000 individuals in endemic areas [11].

Clinical, endoscopic, and imaging examinations cannot distinguish between the pathological types of BCAC; therefore, a diagnosis may be difficult during the initial presentation and would be strictly pathological.

BCAC is composed of the following 2 cell types: smaller basaloid cells with scant cytoplasm and dark nuclei and slightly larger polygonal basaloid cells with eosinophilic cytoplasm [5]. Based on the growth pattern, the tumour can be divided into 4 subtypes: solid, trabecular, tubular, and membranous. The most common histomorphologic pattern is the solid type [12, 13]. Several transitions among these histological types may occur within a single tumour, and the subtype classification is based on the predominant pattern [2, 4].

Published reports on the immunohistochemical aspects of BCAC have discussed the use of dual myoepithelial markers and epithelial markers [14]. A wide range of immunohistochemical diversity is noted between and within BCAC tumours. Immunostaining of the proliferative fraction (Ki-67) is helpful in distinguishing malignancies from their benign counterparts in salivary gland epithelial tumours. The Ki-67 index is typically < 5% in benign tumours and > 10% in malignancies [14].

BCAC is a low-grade malignancy that tends to recur locally [15, 16]. Our literature review revealed that approximately half of patients (4 out of 8, 50.0%) experienced tumour recurrence, and only 1 patient had regional lymph node metastasis. In addition, the mortality rate for this tumour is low. Among 8 patients who were followed up for 0.5–12 years, 2 patients died of disease approximately 4 wears after diagnosis (Table 1).

Treatment of BCAC is well established; however, there are several problems with treatment in clinical practice. Surgical excision with a wide margin to ensure complete tumour removal has been recommended as the mainstay of treatment for BCAC of the major salivary glands. Regional lymph node dissection is indicated only in cases involving cervical lymph nodes [3, 4, 15–17]. BCAC affecting the minor salivary glands are more infiltrative than lesions affecting the major salivary glands [18]. Several surgical approaches have been described, such as an infratemporal approach from the lateral aspect, an antereolateral approach and transpalatal, transmaxillary, and transcervical approaches from the inferior aspect [19]. Radical excision with sufficient healthy margins is difficult mainly due to anatomical boundaries. In addition, postoperative morbidity is considered unacceptable due to the impairment of maxillofacial function in many cases [19].

Radiotherapy is an alternative for inoperable cases after biopsy. Two patients underwent radiotherapy alone due to the anatomical location of the tumour (Table 1). The follow-up indicated that the two cases did not recur or metastasize, and both patients were still alive. The observation period was not long enough (20.9 and 72 months); however, radiotherapy achieved good tumour control.

Most of the previously published studies on BCAC focused on the surgical option and follow-up after surgery, and few studies focused on radiotherapy (Table 1). It is unclear whether radiotherapy is equally as effective as surgery for the management of BCAC. This report contributes to our understanding of the characteristics of BCAC and the treatment of this disease. Radiotherapy may represent an effective method for patients who are not good candidates for surgery. Future studies, such as small-scale clinical trials, are needed to determine whether radiotherapy is effective for patients with inoperable BCAC.

## Conclusions

This case report is the first published description of BCAC that arose from the minor salivary glands in the nasopharynx. BCAC is a low-grade malignancy and tends to recur locally. Wide local excision with confirmation of negative margins has been recommend as the first choice for BCAC of the minor salivary glands. Due to the anatomical location of the nasopharynx, radiotherapy alone was an effective method to treat the tumour. The patient was followed up for 72 months without any evidence of disease. Radiotherapy can achieve good local control and preserve maxillofacial function; therefore, this treatment may be a suitable option for patients who are not good candidates for surgery.

## Abbreviations

AJCC: the American joint committee on cancer
BCAC: Basal cell adenocarcinoma
CK: Cytokeratin
CT: Computed tomography
CTV: Clinical target volume
DOD: Die of disease
F: Female
GTV: Gross tumour volume
IMRT: Intensity-modulated radiation therapy
M: Male
M: Metastasis
MRI: Magnetic resonance imaging
N: Node
NA: Not available
NED: No evidence of disease
NPC: Nasopharyngeal carcinoma
PTV: Planning target volume
RT: Radiation therapy
S: Surgery
T: Tumour

## References

[1] Chen KT (1985). Carcinoma arising in monomorphic adenoma of the salivary gland. Am J Otolaryngol.

[2] Seifert G, Brocheriou C, Cardesa A, Eveson JW (1990). WHO International Histological Classification of Tumours. Tentative Histological Classification of Salivary Gland Tumours. Pathol Res Prac.

[3] Parashar P, Baron E, Papadimitriou JC, Ord RA, Nikitakis NG (2007). Basal cell adenocarcinoma of the oral minor salivary glands: review of the literature and presentation of two cases. Oral Surg Oral Med Oral Pathol Oral Radiol Endod.

[4] Skalova A, Michal M, Simpson RH (2017). Newly described salivary gland tumors. Mod Pathol.

[5] Jayakrishnan A, Elmalah I, Hussain K, Odell EW (2003). Basal cell adenocarcinoma in minor salivary glands. Histopathology.

[6] Chen S, Yang S, Chen X (2015). Basal cell adenocarcinoma of the buccal minor salivary gland with liver metastases. Ann Saudi Med.

[7] Chong VF, Ong CK (2008). Nasopharyngeal carcinoma. Eur J Radiol.

[8] Fonseca I, Soares J (1996). Basal cell adenocarcinoma of minor salivary and seromucous glands of the head and neck region. Semin Diagn Pathol.

[9] Warrick PD, Irish JC, Mancer K, Dardick I, Pynn BR, Gullane P (2000). Basal cell adenocarcinoma: a rare malignancy of the salivary glands. J Otolaryngol.

[10] Jingu K, Hasegawa A, Mizo JE, Bessho H, Morikawa T, Tsuji H, Tsujii H, Kamada T (2010). Carbon ion radiotherapy for basal cell adenocarcinoma of the head and neck: preliminary reportof six cases and review of the literature. Radiat Oncol.

[11] He XY, Liu TF, He SQ, Huan SL, Pan ZQ (2007). Late course accelerated hyperfractionated radiotherapy of nasopharyngeal carcinoma (LCAF). Radiother Oncol.

[12] Baddour HM, Fedewa SA, Chen AY (2016). Five- and 10-Year Cause-Specific Survival Rates in Carcinoma of the Minor Salivary Gland. JAMA Otolaryngol Head Neck Surg..

[13] Loochtan MJ, Shaar M, Pambuccian S, Borrowdale RW (2016). Subglottic Basal Cell Adenocarcinoma. Ann Otol Rhinol Laryngol.

[14] Yamagata K, Oka K, Yoshida H, Yanagawa T, Onizawa K, Yusa H, Ishikawa A, Okada N (2006). Basal cell adenocarcinoma arising from the minor salivary gland in the soft palate: a case report. Pathol Res Pract.

[15] Wilson TC, Robinson RA (2015). Basal cell adenocarcinoma and Basal cell adenoma of the salivary glands: a clinicopathological review of seventy tumors with comparison of morphologic features and growth control indices. Head Neck Pathol.

[16] Ward BK, Seethala RR, Barnes EL, Lai SY (2009). Basal cell adenocarcinoma of a hard palate minor salivary gland: case report and review of the literature. Head Neck Oncol.

[17] Zhan KY, Lentsch EJ (2016). Basal cell adenocarcinoma of the major salivary glands: A population-level study of 509 cases. Laryngoscope.

[18] Cuthbertson DW, Raol N, Hicks J, Green L, Parke R (2015). Minor salivary gland basal cell adenocarcinoma: a systematic review and report of a new case. JAMA Otolaryngol Head Neck Surg.

[19] Wei WI, Sham JS (2005). Nasopharyngeal carcinoma. Lancet.

